# Survey data on global shipping lines assessing factors of container port competitiveness

**DOI:** 10.1016/j.dib.2020.105444

**Published:** 2020-03-19

**Authors:** Adam Kaliszewski, Arkadiusz Kozłowski, Janusz Dąbrowski, Hanna Klimek

**Affiliations:** a Department of Maritime Transport and Seaborne Trade, Faculty of Economics, University of Gdańsk, ul. Armii Krajowej 119/121, 81-824 Sopot, Poland; b Department of Statistics, Faculty of Management, University of Gdańsk, ul. Armii Krajowej 101, 81-824 Sopot, Poland

**Keywords:** Competitiveness, Container terminals, Ports, Strategy, Adaptability, Governance, Shipping lines, Linkedin

## Abstract

Competitiveness of seaports is a matter of interest not only to the economists, but also businesses, governments and international organizations. This data article provides quantitative data from the survey research on factors of competitiveness of container ports as perceived by shipping lines.

The data was collected from around the world using an online questionnaire distributed through LinkedIn. The spatial dispersion of respondents corresponds approximately to the structure of global maritime container trade. The data provides full responses from 120 respondents. Each respondent assessed the importance of 20 predefined competitiveness factors on a scale of 1 (least important) to 10 (most important). For each respondent two additional characteristics are known, the location (continent) and the size of the company for which he/she works, measured by the number of employees.

The data were used for a research article to determine the ranking of competitiveness factors for container ports entitled “Key factors of container port competitiveness: A global shipping lines perspective” [1]. The data can be used for another research to uncover relationships between factors of competitiveness (through e.g. factor analysis, cluster analysis), both for the whole world and for groups by continents or the size of the company.

Specifications table

**Table:** 

Subject	Business and international management
Specific subject area	Container ports management
Type of data	Table
How data were acquired	Online survey via LinkedIn (https://containers.limequery.com/75862?lang=en)
Data format	Raw Filtered
Parameters for data collection	The respondents were given 20 competitiveness factors of maritime container terminals to which they were asked to assign points from 1 to 10, where 1 means a factor is completely unimportant and 10 means it is very important for the respondent.
Description of data collection	The population of potential respondents was defined as managers and directors, who were: - current employees of shipping lines being listed on Alphaliner's TOP 100 as of 9th April 2019, - members of the LinkedIn social network at the same time. A brief questionnaire in English language was distributed to these group members by invitation, partially handled by an automated invitation tool based on precise respondent profile from the prepared list and partially in a manual process. The whole proccess was similar to snowball sampling method, and took 2.5 months.
Data source location	Global
Data accessibility	Repository name: Mendeley Data Data identification number: DOI: 10.17632/vn5ym7n7kb.1 Direct URL to data: https://data.mendeley.com/datasets/vn5ym7n7kb/1
Related research article	A. Kaliszewski, A. Kozłowski, J. Dąbrowski, H. Klimek, Key factors of container port competitiveness: A global shipping lines perspective, Marine Policy, in press

## Value of the data

• The data are unique because they present opinions of managers and directors of shipping lines operating in different locations around the world, and the representation of respondents is approximately proportional to the structure of container trade around the world.
• The data can be useful for researchers dealing with the competitiveness of container ports and for port managers in order to build a long-term action strategy.
• The data can be used to uncover relationships between factors of competitiveness (through e.g. factor analysis, cluster analysis), both for the whole world and for groups by continents or the size of the company.

## 1 Data description

The data consist of one raw data file of 120 observations (responses) and 22 variables. The variables (columns) from 1 to 20 represent the 20 competitiveness factors, and they are as follows:

1. Terminal charges (THC), price, rebates and other financial incentives
2. Scope of terminal services and logistic value added services
3. Level of container terminal service quality (speed, reliability, availability, security, non-discriminatory access, eco-friendliness)
4. Port community system (serving port clients, other stakeholders as well as inside container terminal)
5. Terminal's ability to serve mega container vessels (TEUs + 18k)
6. Intermodal transport availability in the container terminal (by rail, inland waterways and roads)
7. Private ownership of terminal
8. Partial ownership of a terminal by shipping lines
9. Terminal's adaptability to the changing market environment
10. Level of harmony in management-labor-government relationships (no strikes, conflicts and others)
11. Corporate Social Responsibility (incl. business ethics, respect of natural environment and involvement with local communities)
12. Terminal operations respecting natural environment protection laws
13. Port's reputation, public relations and marketing
14. Port's nautical accessibility
15. Maritime connectivity (frequency of shipping services)
16. Hinterland connection (road and rail networks, inland waterways)
17. Port authority charges, price and pricing strategies
18. Fast customs and admin clearance of cargo, incl. port's regulations and customary duties
19. Shipping lines concentration level (M&A, alliances) and changes in shipping lines' preferences
20. Supportive government active in promoting ports and logistics transport policies

These first 20 variables take whole values from 1 – meaning the factor is least important for the respondent, to 10 – meaning the factor is most important for the respondent.

Two more variables are as follows:

21. Size – number of people employed by the company for which the respondent is working. Possible answers are:
  a. Less than 10 persons
  b. 10 to 49 persons
  c. 50 to 249 persons
  d. 250 or more persons
22. Continent – the continent where the respondent work (the respondents were asked to choose in which country they work, which due to confidentially reasons was aggregated to the level of continents). Possible answers are:
  a. Asia
  b. Europe
  c. South America
  d. North America
  e. Australia
  f. Africa

Any item nonresponses were left blank.

## 2 Experimental design, materials, and methods

The list of potential respondents was prepared following two criteria. The first was shipping lines membership pursuant to Alphaliner's TOP 100 as of 9th April 2019, which includes active vessels in container liner services business. The number of ships for various Maersk Group companies, as well as other lines, was consolidated and presented as one by Alphaliner [2]. In the second step, a group of senior managers and directors, who were current employees of these companies and members of the LinkedIn social network at the same time, was selected. Based on Forbes assessment LinkedIn is “the most advantageous social networking tool available to job seekers and business professionals today” [3]. Other social media networks are less business-oriented, thus it is harder to contact target group members. LinkedIn network for an individual consists of first, second and third level connections, group memberships, while direct contact with members outside of a user's network requires paid invitations, known as InMails. Professionals outside of a user's network are marked as inaccessible “LinkedIn members”. Thus, it is not technically possible to reach all respondents at once. Instead, a method similar to the snowball sampling method was used, as in the earlier case of Facebook survey [4]. The cost of all online search tools, survey creation and hosting tools per usable response was USD 1.10. This excludes time and effort of the main author during 2.5 months survey timing during the intensive iterative process of sending invitations (semi-automated way using an online invitation tool), answering queries from potential respondents (those who accepted the invitation), actual respondents (those who opened the survey and fully/partially completed it). The iterations were needed as, in practice, only 1st and 2nd level LinkedIn members responded to the survey, while none by paid InMail's, which potential respondents ignored. Thus, the number of “my network” 1st level connections snowballed 6 times during the iterative process.

The questionnaire was written in English and was hosted on LimeSurvey (https://www.limesurvey.org/).

Out of all invitations sent, 210 persons attempted the survey, with 120 full responses recorded. The overall response rate for this survey was 8% among persons who have accepted the invitation. The data made available are filtered to present only the full, useable responses.

Ethical considerations: there were no ethical issues associated with this survey because the responses were fully anonymous (responses were not linked with respondents’ LinkedIn accounts) and the topic of the survey was far from sensitive.

## Conflict of Interest

The authors declare that they have no known competing financial interests or personal relationships that could have appeared to influence the work reported in this paper.

## Appendix Supplementary materials

Image, application 1
